# Management of extravasations of enfortumab vedotin and pembrolizumab: a case report and mini review of the current literature

**DOI:** 10.1007/s00432-026-06646-y

**Published:** 2026-10-16

**Authors:** Hendrik Dinkel, Konstantin E. Seifert, Martin Bögemann, Andres J. Schrader, Katrin Schlack

**Affiliations:** Department of Urology, University Hospital Münster, Albert-Schweitzer-Campus 1, GB A1, 48149 Münster, Germany

**Keywords:** Urothelial cancer, Enfortumab vedotin, Antibody-drug conjugate, Pembrolizumab, Extravasation, Case report

## Abstract

Following the EV-302/KEYNOTE-A39 study, the combination therapy of enfortumab vedotin and pembrolizumab (EV-P) has been established as a new first-line treatment for metastatic urothelial carcinoma. With increasing use, the documentation and publication of unexpected side effects and complications are essential to systematize procedures. We report on a 56-year-old patient who experienced an extravasation of EV-P during the first cycle due to dislocation of the port catheter from the cephalic vein. Extensive subcutaneous necrosis of the infraclavicular region required surgical debridement and application of a vacuum-assisted closure system, followed by secondary wound healing. The wound healed with a residual scar but no functional impairment or persistent pain. The therapy was resumed via a peripheral venous catheter, and the patient achieved a deep partial response. To our knowledge, this represents the first reported case of central venous EV-P extravasation (CTCAE grade 3) requiring surgical therapy. Because of its cytotoxic payload, EV in particular may pose a high risk of local complications in the context of extravasations.

## Introduction

Enfortumab vedotin (EV) is an antibody-drug conjugate (ADC) that targets Nectin-4, a glycoprotein expressed on the surface of urothelial carcinoma (UC) cells. Nectin-4 thus represents a target for selective therapy. Binding of the antibody component enfortumab to Nectin-4 induces receptor-mediated endocytosis and lysosomal degradation in the cytoplasm. The cytotoxic payload monomethyl auristatin E (MMAE) is released and inhibits the microtubule polymerization, leading to apoptotic cell cycle arrest (Doronina et al. 2003). This mechanism provides an approach to deliver a potent cytotoxic drug to tumor cells while potentially minimizing side effects.

Pembrolizumab is more extensively used in immuno-oncology to enhance response of the immune system against various types of cancer. It specifically targets the programmed cell death protein 1 (PD-1) receptor, which is expressed on activated T-lymphocytes. Under physiological conditions, T-lymphocytes are suppressed by interaction of PD-1 with its ligands PD-L1 and PD-L2. Thereby, a certain immune tolerance is enabled to avoid autoimmune reactions. In many cancers, tumor cells or the tumor microenvironment overexpress PD-1 ligands to facilitate immune escape. By inhibiting the PD-1/PD-L1 interaction, pembrolizumab reactivates the T-cell response and prevents tumor immune escape (Bellmunt et al. 2017; Zhang et al. 2016). This mechanism has been demonstrated in various tumor types, including renal cell carcinoma (Makker et al. 2022; Choueiri et al. 2024), squamous cell carcinoma (Tamimi et al. 2023), malignant melanoma (Patel et al. 2023), non-small cell lung cancer (Garon et al. 2015) and UC (Bellmunt et al. 2017).

The targeted cytotoxic effect of EV and the immune potentiation by pembrolizumab have recently shown practice-changing results in the treatment of metastatic UC (mUC) in the phase III EV-302/KEYNOTE-A39 study (Powles et al. 2024). The combination therapy has become the treatment of choice in first-line therapy of mUC and exemplifies the trend towards combination therapies in immuno-oncology.

With the increasing use of EV and pembrolizumab (EV-P), rare adverse events and complications are increasingly recognized. Extravasations, defined as a leakage of drugs from the blood vessel into the tissue, are rare and potentially serious (Perez Fidalgo et al. 2012). Here, we present the first published case of a CTCAE (version 6.0) grade 3 adverse event in form of a central extravasation resulting from port dislocation from the right cephalic vein.

The patient provided written informed consent for the publication of the clinical data and any images included in this manuscript. For the review, a systematic PubMed (MEDLINE) literature search was conducted using Medical Subject Headings (MeSH) and keywords related to (1) enfortumab vedotin and pembrolizumab and synonyms or abbreviations and (2) extravasation and related terms. Only peer-reviewed articles published in English before August 3, 2026, were considered.

## Case presentation

A 56-year-old male patient was diagnosed with UC of the right distal ureter with lymphatic and bone metastasis. First-line treatment with EV 1.25 mg/kg (d1 and d8) and pembrolizumab 200 mg q3w was initiated according to the EV-302/KEYNOTE-A39 protocol (Powles et al. 2024). A port system had been implanted in the right cephalic vein three weeks earlier and had not been used before. The patient was asymptomatic in this respect. After informed consent was obtained from the patient, the port chamber was punctured routinely without any problems. We started the infusion of the combination therapy, and the patient did not notice any irritation, pressure or pain during the infusion. After the total amount of 300 mL had been infused, a local swelling of the infraclavicular region was observed by the medical staff. Due to the obvious suspicion of an extravasation, the region was immediately cooled and, after consultation with general surgery, infiltrated subcutaneously with hyaluronidase. The port needle was removed after aspiration was not possible. The chest X-ray revealed a dislocated port catheter from the cephalic vein with no evidence of pneumothorax, fluid collection or pleural effusion. After hospitalization of the patient and continuation of the local measures, extensive redness and detachment of the upper skin layers developed in the following days. Also, the patient developed a local sensation of pressure, but no fever, pain or functional impairment of the right arm. The treatment was coordinated by an interdisciplinary team of urologists, general surgeons, wound and ostomy nurses and dermatologists.

**Fig. 1:**
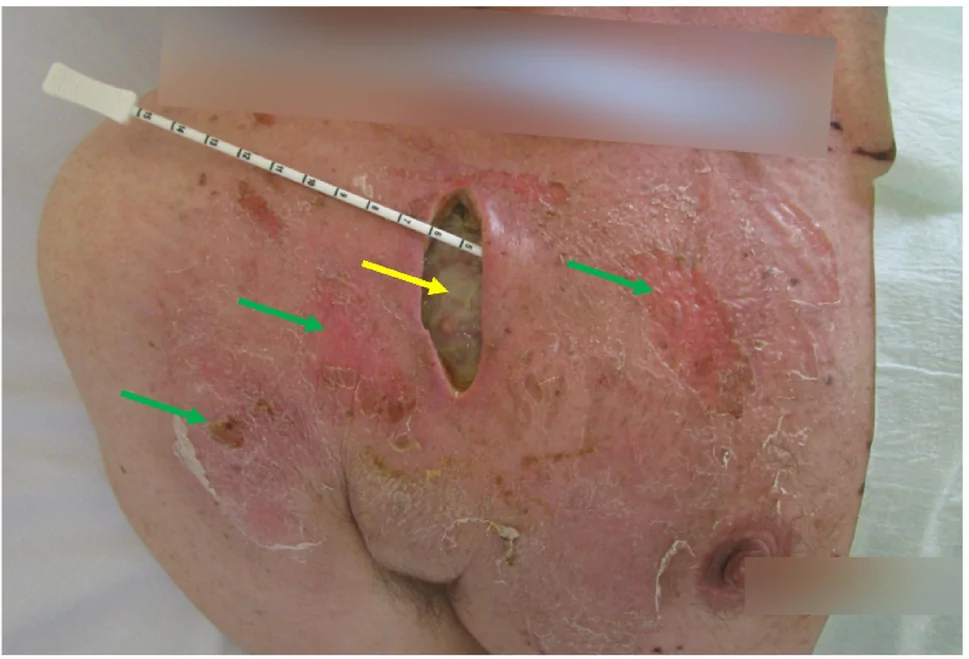
Right infraclavicular region on day 13 after central venous EV-P extravasation. The image shows extensive deep fatty tissue necrosis (yellow arrow) surrounded by bullous dermatitis (green arrows). EV-P, enfortumab vedotin and pembrolizumab.

**Fig. 2:**
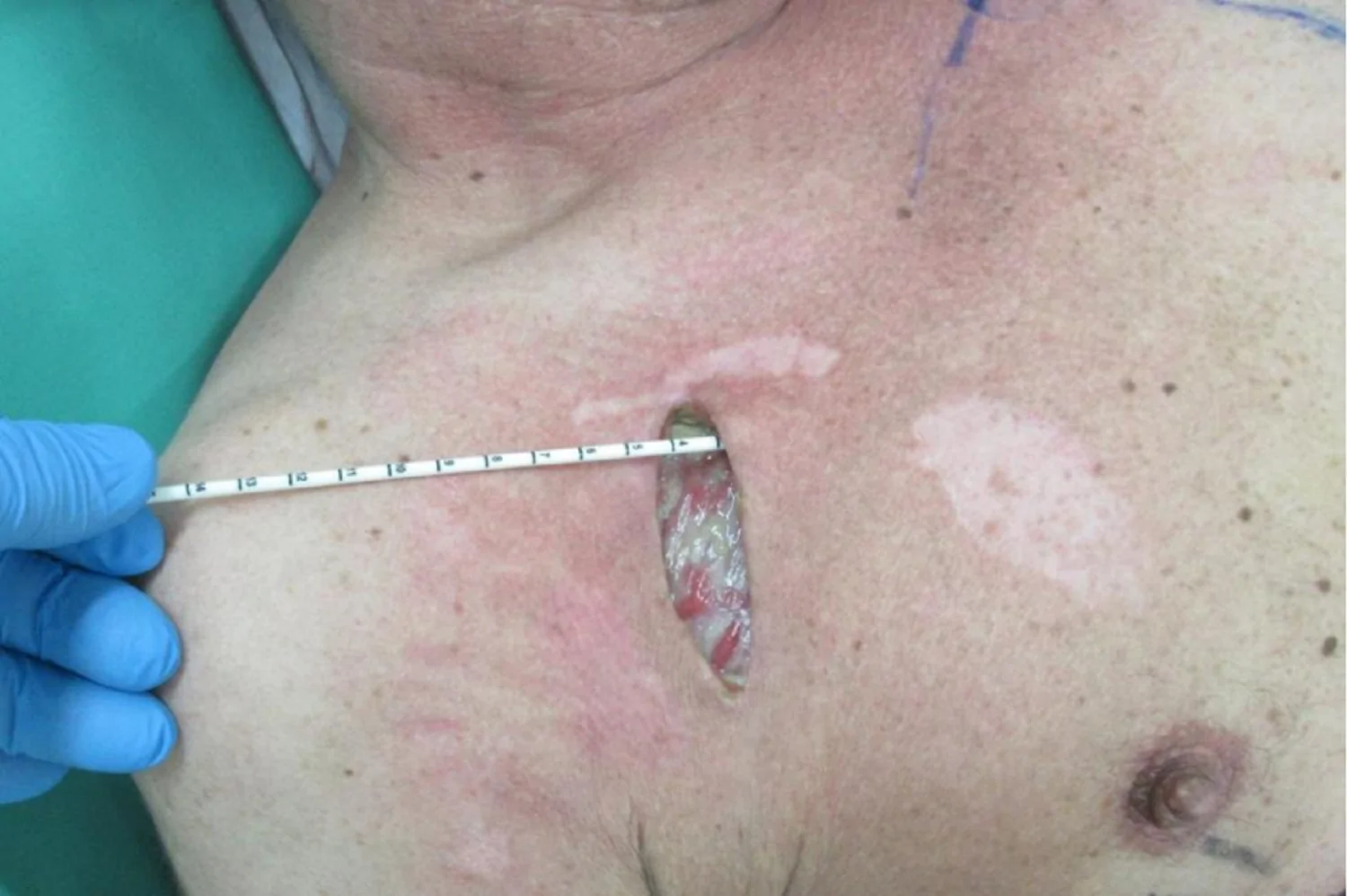
Right infraclavicular region on day 29 after central venous EV-P extravasation. The skin is now intact, and the previously necrotic fatty tissue is regressing in the context of secondary wound healing (yellow arrow). Compared with Fig. 1, the surrounding inflammatory dermatitis is markedly reduced (green arrows). EV-P, enfortumab vedotin and pembrolizumab.

**Fig. 3:**
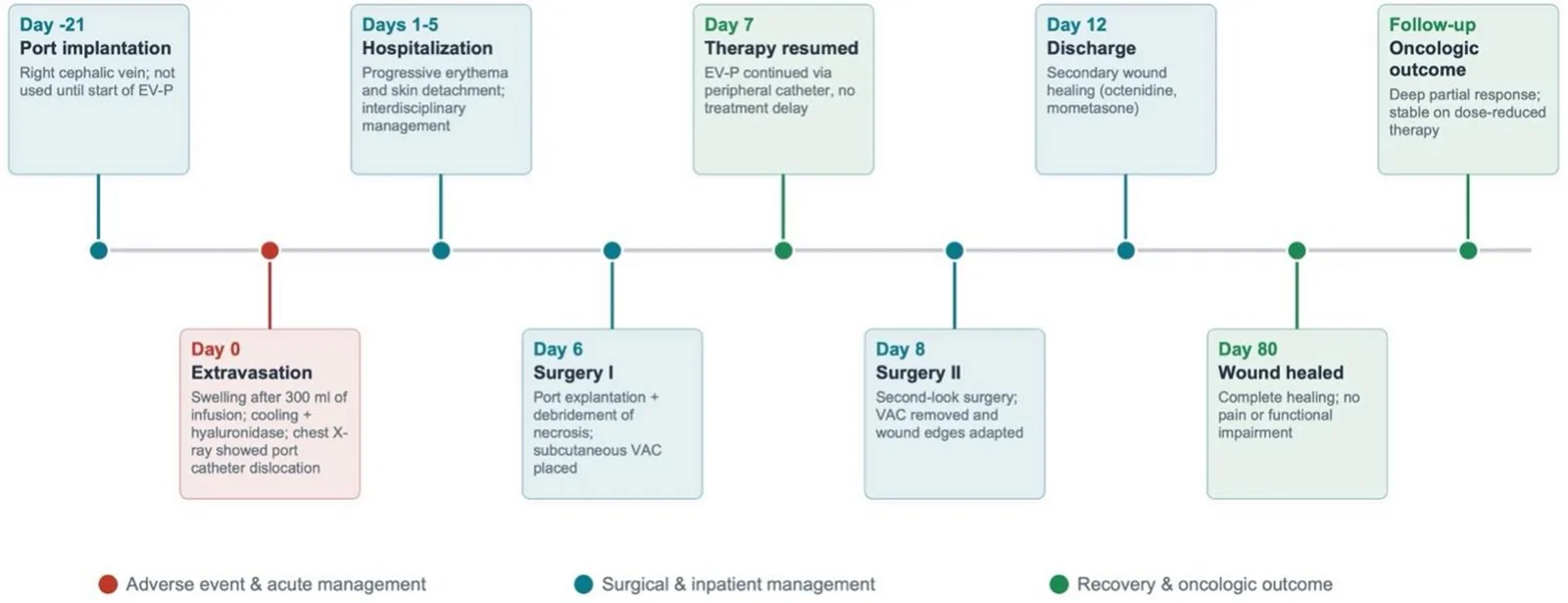
Timeline of the clinical course from port implantation to complete wound healing and oncologic follow-up, day 0 = day of extravasation. EV-P, enfortumab vedotin and pembrolizumab; VAC, vacuum-assisted closure.

On day 6 after the extravasation, general surgeons explanted the port system. No complications occurred during the procedure. However, extensive fatty tissue necrosis of the infraclavicular region was observed, so wound debridement was performed and a subcutaneous VAC (vacuum-assisted closure) system was applied. After two days, the VAC was removed in second-look surgery, and the wound edges were approximated. Dermatologists recommended application of octenidine daily and the surrounding dermatitis was treated with mometasone and 3 M™ Adaptic™ as part of the secondary healing process. The patient was discharged on day 12 after extravasation and day 6 after removal of the port system (Figs. 1, 2 and 3). During follow-up, the wound healed completely within 80 days with no residual open areas, persistent pain or functional impairment. The residual scar causes no functional or cosmetic problems.

As therapy with EV was resumed on day 7 after the extravasation, no treatment delay occurred. Further administrations of EV-P were given via a peripheral venous catheter without complications. The patient subsequently achieved a deep partial response (RECIST 1.1) and is currently stable under dose-reduced therapy.

## Discussion

Depending on the administered drug, extravasations are rare but potentially severe complications in the systemic treatment of patients with metastatic cancer. Antineoplastic agents are classified as irritants, vesicants or neutral depending on their potential to harm the surrounding tissue. Irritants usually cause localized pain or inflammation, while vesicants may result in necrosis and blister formation (Kreidieh et al. 2016; Ener et al. 2004). The guidelines for the management of extravasation injuries of the European Society for Medical Oncology (ESMO) and European Oncology Nursing Society (EONS) recommend treatment based on the specific drug. General measures include immediate termination of the infusion, careful aspiration of as much of the extravasated fluid as possible (e.g. using a 10 mL syringe) and removal of the intravenous catheter or port needle. It is also critical to ensure complete documentation, including demarcation of erythema or swelling and precise photographic and written records. Additionally, elevation of the affected body part, subcutaneous administration of specific antidotes and the application of cold or warm compresses can provide supportive care.

In the case presented, a CTCAE grade 3 extravasation occurred with the vinca alkaloid-like substance MMAE. For vinca alkaloids and taxanes, the ESMO/EONS guidelines generally recommend the application of moderate warmth for 20 min four times daily to support absorption through local vasodilation. In this case, however, cold compresses were applied immediately after extravasation as a general first-line measure to limit local swelling and spread. Cold application causes vasoconstriction and may confine the extravasate, which is appropriate for other vesicants (e.g. anthracyclines). If initial cooling may have contributed to the extent of necrosis by retaining the cytotoxic payload within the tissue remains uncertain as there is no supporting evidence. However, the case underscores that, for MMAE-based ADCs, warm compresses with hyaluronidase should be preferred over cold application (Perez Fidalgo et al. 2012; Wengstrom et al. 2008).

In three previously published case reports of extravasations of EV through peripheral venous access in the antecubital fossa, management followed the same protocol, while surgical intervention was not required. These cases showed local skin reactions, including erythema, exfoliation of the skin and blister formation. Similar to our case, the symptoms developed over several days and gradually resolved with conservative management (Grant et al. 2023; Pulavarty et al. 2024). In one case of EV extravasation, treatment with subcutaneous hyaluronidase using the “pin cushion” technique and the application of intermittent heat resulted in no skin lesions, which underscores the importance of adequate acute therapy (Pipitone et al. 2026). Additionally, there are reports of extravasations of MMAE conjugated to antibodies targeting CD30 (brentuximab), CD79b (polatuzumab) and tissue factor (tisotumab) (Son et al. 2024; Hoffmann et al. 2020; Rivasi et al. 2024; Kim et al. 2022; Toulmin et al. 2025). In these examples, extensive necrosis occurred, along with the partial development of peripheral neuropathy (Table 1).

**Table 1:** Published cases of extravasations of MMAE-containing antibody-drug conjugates *ADC*, antibody–drug conjugate; *EV*, enfortumab vedotin; *IV*, intravenous; *MMAE*, monomethyl auristatin E

ADC (target)	Patient & malignancy	Venous access & onset	Clinical presentation	Management	Outcome	Reference
Enfortumab vedotin (Nectin-4)	59-year-old woman and 63-year-old man with metastatic urothelial carcinoma	Peripheral IV, right antecubital fossa; after a few hours	Forearm erythema, edema and severe pain; blister formation; fever and altered mental status; later bulla with central erosion and desquamation	Conservative; IV antibiotics for presumed cellulitis (clindamycin or vancomycin + ceftriaxone); warm compresses; no surgery	Clinical improvement and discharge; both patients resumed EV without further adverse events	Grant et al. 2023. J Oncol Pharm Pract 29(7):1789–1792
Enfortumab vedotin (Nectin-4)	59-year-old man with metastatic urothelial carcinoma	Peripheral IV, right antecubital fossa; progressive over one week after the infusion	Desquamating rash with epidermal sloughing of the forearm, exposing papillary dermis; minimal tenderness	Conservative wound care; bismuth-petrolatum gauze wrap, changed daily	Skin findings remained stable; no surgical intervention	Pulavarty et al. 2024. JAAD Case Rep 46:15–17
Enfortumab vedotin (Nectin-4)	79-year-old woman with metastatic urothelial carcinoma	Peripheral IV	Burning pain and edema; no skin alterations	Subcutanous hyaluronidase, warm compresses	No pain oder skin damage	Pipitone et al., 2025. Eur J Hosp Pharm; ejhpharm-2024-004323
Brentuximab vedotin (CD30)	80-year-old man with CD30 + lymphoma	Peripheral IV; onset 1 day after extravasation; progression over 1 week	Circumscribed blistering lesion with local toxicity, accompanied by demyelinating peripheral neuropathy	Not reported	Full resolution within 2 months	Hoffmann et al. 2020. J Eur Acad Dermatol Venereol 34(10):e626–e628
Brentuximab vedotin (CD30)	87-year-old man with mycosis fungoides and CD30 + large cell transformation	Peripheral IV, left antecubital fossa; progress over 1 week	Erythema at the site leading to painful superficial epidermal necrosis, desquamation and patchy erosions; no neuropathy	Infusion stopped; cold compress; conservative	Full resolution within 2 months	Kim et al. 2022. Dermatol Ther 35(9):e15751
Brentuximab vedotin (CD30)	Patient in his 70s with anaplastic large cell lymphoma	Peripheral IV, left antecubital fossa	Initially rash and edema, progressing to extensive epidermal necrosis	Cold compress; antibiotics with amoxicillin and clavulanic acid, fusidic acid cream, diprosone cream, covered with paraffin gauze	Full resolution (> 2 weeks)	Rivasi et al., 2025. Eur J Hosp Pharm 33:94–97
Tisotumab vedotin (Tissue factor)	62-year-old woman with metastatic squamous cell cervical carcinoma	Peripheral IV, right antecubital vein; symptoms from day 5	Day 5 mild erythema and pain; progression to pustules and skin sloughing by day 12, prompting hospital admission	Initial topical clindamycin + cold compresses; on admission multidisciplinary consultation; topical clobetasol 0.05%, Mepitel One Silicone^®^ and PolyMem Silver^®^	Gradual resolution over 11 weeks	Son et al. 2024. Gynecol Oncol Rep 56:101525
Polatuzumab vedotin (CD79b)	60-year-old man with diffuse large B-cell lymphoma	Peripheral IV, right arm; symptoms on day 6	Swelling, pain and erythema progressing to enlarging bulla and tenderness	Local wound care with plain petrolatum, 3% bismuth tribromophenate petrolatum impregnated gauze	Complete healing within 4 weeks	Toulmin et al. 2025. Leuk Lymphoma 66(6):1169–1171

The precise mechanism of necrosis associated with EV or specifically the payload MMAE is not fully understood. The principal contributor is presumably MMAE, which inhibits tubulin polymerization, arrests cells in mitosis and induces cell death. Because MMAE is small and lipophilic, it crosses cell membranes as soon as it is liberated from its antibody carrier and is distributed throughout the surrounding extracellular space. As adipocytes and dermal stromal cells do not require Nectin-4 expression to be susceptible to membrane-permeable MMAE, this mechanism provides a potential explanation for the tissue necrosis that dominated the clinical picture (Staudacher and Brown 2017). A Nectin-4 dependent mechanism may have potentiated the cutaneous component, since the target is expressed not only by UC cells but also physiologically by epidermal keratinocytes. EV binds to these Nectin-4 positive healthy cells in vivo. Internalization and intracellular MMAE delivery are the accepted basis of EV-associated dermatologic toxicity. Histopathology of such lesions typically shows extensive keratinocyte apoptosis (Lacouture et al. 2022). We therefore hypothesize that the antigen-independent payload toxicity to the adipose tissue led to extensive necrosis and the antigen-directed toxicity to Nectin-4 expressing keratinocytes might have potentiated the dermal injury, leading to erythema, blistering and epidermal detachment. This combined mechanism is supported by the high overall rate of cutaneous toxicity reported for EV (Lacouture et al. 2022), however, there is no conclusive evidence.

As a PD-1 immune-checkpoint inhibitor, pembrolizumab is not a vesicant and would not be expected to cause direct cytotoxic tissue necrosis after extravasation. However, it enhances T-cell activity and is associated with immune-related skin reactions (Rodriguez-Alarcon and Conde-Estevez 2021; Bolzacchini 2019). An immune-mediated component occurring in addition to the primary payload injury cannot be excluded, although we consider MMAE-driven cytotoxicity the principal driver in this case.

We emphasize that this explanation is speculative. No biopsy or Nectin-4 immunohistochemistry of the affected tissue was performed and the relative contributions of antigen-independent payload toxicity, Nectin-4 directed keratinocyte injury and immune-mediated inflammation cannot be established from a single case.

As the use of ADCs increases, experience with extravasations will also grow. To date, many ADCs have not been categorized regarding their harmful effects in the event of extravasation. We hypothesize that MMAE may act as a vesicant upon extravasation as do other tubulin-targeting agents, such as docetaxel and paclitaxel (Barbee et al. 2014). We suggest that the management should be guided by the payload of the ADC, while also considering the toxicity profile of the antibody.

Finally, the prevention of drug extravasations in the context of oncological therapy remains the most important measure. First, careful attention should be given to safe peripheral or central venous access. Ideally, peripheral access should not be placed in the antecubital vein or the dorsal hand veins, but rather in the ventral forearm. All physicians and nurses involved must be well-versed in the management of extravasations and receive regular training. Management of central versus peripheral extravasations may differ, as the large area of tissue affected with central access may increase the risk of extensive necrosis. In this case, a conservative approach was initially pursued, followed by a debridement after the port removal.

This case underlines the need for an interdisciplinary approach after extravasation of cytotoxic substances. All events should be documented and published to optimize the treatment of EV extravasations and update current guideline recommendations.

## Conclusion

This case highlights that extravasations of EV-P, particularly via central venous access, may lead to severe tissue damage that may require surgical management. Based on this single observation, the cytotoxic payload MMAE may exhibit vesicant-like properties, although this remains to be confirmed. Careful handling of vascular access and early interdisciplinary management appear advisable to minimize complications. Further reports are needed to characterize the risk profile of EV-P extravasations before evidence-based management guidelines can be established.

## Key learning points

- Based on this clinical case, we hypothesize that EV/MMAE may show vesicant-like properties in the context of extravasations.
- MMAE-based ADCs are formally classified, so extravasation management should be based on the payload rather than on the antibody.
- Initial local treatment should follow the ESMO/EONS guidelines: stop infusion, attempt aspiration, remove needle, elevate limb and apply subcutaneous hyaluronidase with dry warmth (20 min, four times daily).
- Central venous extravasation may be more severe than peripheral events and large infused volumes may lead to extensive fat necrosis requiring surgical intervention.
- Port patency must be confirmed before every vesicant infusion; peripheral infusion is preferably given via the ventral forearm rather than the antecubital fossa or dorsal hand veins.
- Structured interdisciplinary follow-up is essential while systemic therapy can often be resumed via alternative access without treatment delay.

## Data Availability

Not applicable.
